# A One Health comparative genomic assessment of antimicrobial-resistant *Escherichia coli* in dairy farms in western Canada

**DOI:** 10.1128/aem.01905-25

**Published:** 2026-01-27

**Authors:** Cassandra Klaas, Shawn Hoogstra, David Mahoney, Mark Lubberts, Siyun Wang, Robin Richter, Kasia Dadej, Audrey Charlebois, Daniella Rizzo, Richard J. Reid-Smith, Rhiannon L. Wallace

**Affiliations:** 1 Agassiz Research and Development Center, Agriculture and Agri-Food Canada 98645, Agassiz, British Columbia, Canada; 2 Department of Community Health and Epidemiology, Faculty of Medicine, Dalhousie University 12361, Halifax, Nova Scotia, Canada; 3 Faculty of Computer Science, Dalhousie University 153020, Halifax, Nova Scotia, Canada; 4 Summerland Research and Development Center, Agriculture and Agri-Food Canada 98646, Summerland, British Columbia, Canada; 5 Food, Health, and Nutrition, University of British Columbia 8166, Vancouver, British Columbia, Canada; 6 Ottawa Research and Development Center, Agriculture and Agri-Food Canada 98674 , Ottawa, Ontario, Canada; 7 National Microbiology Laboratory, Public Health Agency of Canada 41687, St-Hyacinthe, Quebec, Canada; 8 Centre for Foodborne, Environment and Zoonotic Infectious Diseases, Public Health Agency of Canada 41687, Guelph, Ontario, Canada; Centers for Disease Control and Prevention, Atlanta, Georgia, USA

**Keywords:** antimicrobial resistance, *Escherichia coli*, extended spectrum beta-lactamase, phylogenetics, One Health, whole-genome sequencing

## Abstract

Antimicrobial resistance (AMR) is a growing global public health concern affecting animals, humans, and the environment. Given its ubiquity, *Escherichia coli* may play a key role in the dissemination of AMR across these domains. Peri-urban regions where urban and rural systems intersect present unique challenges for controlling AMR. Despite this, limited data are available on AMR dissemination across the One Health continuum in peri-urban settings such as the Fraser Valley region of Canada. This study adopted a One Health approach to assess associations in AMR traits between *E. coli* from dairy production systems, nearby natural environments, and peri-urban communities within the same geographic location and timeframe. Over 1,000 isolates were recovered, and 421 were whole-genome sequenced to determine the presence of antimicrobial resistance genes (ARGs), plasmids, and virulence genes and to assess genomic relatedness. Findings revealed that AMR was not widespread: 17.8% of isolates carried at least one ARG, 10.2% were classified as multidrug-resistant, and 9.5% carried beta-lactamase genes. Phylogenomic analysis revealed high genomic diversity, with isolates belonging to 174 different sequence types (STs), including clinically important ST131 and ST10. Pairwise single-nucleotide polymorphism (SNP) comparisons identified 207 isolate pairs differing by ≤100 SNPs, indicating early-stage cross-domain movement of AMR. Overall, the findings from this study show that the prevalence of AMR *E. coli* is low, but there is evidence of transmission between animals and the environment, highlighting the importance of proactive, integrated surveillance and mitigation strategies to reduce the risk of future AMR dissemination across the One Health continuum.

**IMPORTANCE:**

Antimicrobial resistance (AMR) is a global public health concern that spans all three One Health domains (humans, animals, and the environment). *Escherichia coli* is present in humans, animals, and environmental sources—its ubiquity makes it an ideal organism to study AMR hotspots and transmission pathways across the One Health continuum. While surveillance of AMR in agricultural settings is increasing globally, little is known about transmission pathways in peri-urban agriculture areas where there is a high density of livestock farming in close proximity to residential communities. To identify potential AMR hotspots and transmission routes, this study investigated the occurrence and genomic relatedness of generic *E. coli* in the Fraser Valley region of British Columbia, a highly diverse agricultural region in western Canada. Our findings expand current knowledge by suggesting that early-stage transmission of AMR is occurring between the human, animal, and environmental sectors of the One Health triad, highlighting areas for improved resistance mitigation to prevent widespread dissemination.

## INTRODUCTION

*E. coli* is ubiquitous and is found in a variety of environments, such as soil, water, and the gastrointestinal tract of many mammals, including humans and food-production animals (1, 2). While most strains of *E. coli* are harmless or even beneficial to their hosts, some strains are pathogenic and can cause disease or infection (1, 3). Many *E. coli*-associated diseases and infections are treatable with antimicrobials, such as penicillins, cephalosporins, and monobactams (4). However, resistance to these antimicrobials has become increasingly common (5). *E. coli* and other bacteria can achieve antimicrobial resistance (AMR) through several mechanisms, such as efflux pumps that pump antimicrobials out of the cell, enzymatic degradation of antimicrobials, modification of antimicrobial targets, and changes in membrane permeability (5). In Canada alone, AMR is responsible for over 14,000 deaths each year (6).

Antimicrobials are often used in agricultural settings for the treatment and prevention of different infections in farm animals, such as abscesses and respiratory diseases (2). Global antimicrobial use in food production animals is expected to increase by 67% in 2030 compared to 2010, highlighting the concern of food production animals and agricultural settings playing a major role in the dissemination of AMR (7). Presently, limited baseline data are available on AMR in intense livestock farming regions located in peri-urban settings such as the Fraser Valley. The drivers of AMR dissemination often differ between urban and rural areas. In urban settings, high population density and healthcare-associated factors are key contributors, whereas in rural regions, agricultural practices play a more prominent role (8). The close proximity of human populations, livestock operations, and natural environments in such regions can increase interactions among the One Health domains, thereby facilitating cross-domain AMR transmission. As a result, peri-urban areas may pose a distinct risk for AMR spread due to the convergence of urban and agricultural influences.

Due to the ubiquitous nature of *E. coli*, there are many reservoirs across the One Health continuum where *E. coli* and its genetic determinants can persist and propagate (1), including livestock animals such as dairy cows, humans, soil, and surface water (3, 8). Unfortunately, despite recognition of AMR as a global public health concern (9), data gaps regarding the role of livestock operations in the dissemination of AMR persist (10, 11). The dairy industry is an important agricultural sector within Canada, particularly in British Columbia. In 2024, Canada had 9,256 dairy farms, with 426 located in British Columbia (12–14). Over 75% of the province’s milk production occurs in the Fraser Valley region of British Columbia (13), which is home to 324,005 Canadians (15). This agriculturally diverse peri-urban setting presents unique challenges for AMR surveillance and mitigation due to the high density of dairy farms situated alongside urban and natural environments.

The overarching goal of this study was to identify associations in AMR traits among *E. coli* isolates recovered from dairy production systems, nearby natural environments, and peri-urban communities within the same geographic location and timeframe. This research aims to address current knowledge gaps by assessing whether transmission of AMR *E. coli* is occurring across the One Health continuum from dairy farms to adjacent environments and communities. This study recovered *E. coli* isolates from human, animal, and environmental sources, representing all three One Health domains. Whole-genome sequencing (WGS) was used to assess the occurrence of AMR determinants, including ARGs, plasmids, and virulence factors, and to evaluate the potential risk of AMR transmission across the One Health continuum.

## MATERIALS AND METHODS

### Study site and sample collection

Manure and environmental samples were collected seasonally (summer, fall, winter, and spring) from three conventional and three organic dairy farms and surrounding environments in the Fraser Valley region of British Columbia over a 2-year period (2022–2024). A subset of farms enrolled in the Canadian Dairy Network of Antimicrobial Stewardship and Resistance (CaDNetASR) (16) surveillance program was recruited via in-person farm visits to participate in this study. Housing conditions were typical for western Canadian dairy herds with cows housed in free stalls in open-air barns with concrete floor pens with wood shavings or sand for bedding. The size of the herd ranged from 140 to 700 cows, with the number of milking cows ranging from 65 to 270. Cows were typically housed based on age group and whether they were milking or dry cows. Four of the six farms used robotic milking systems. The three organic farms allowed their milking and dry cows to have access to pasture during the summer months, while the three conventional farms housed their entire herd indoors year-round.

Manure sampling was conducted according to a protocol approved by the Lethbridge Research & Development Centre Animal Care Committee, Agriculture and Agri-Food Canada (Protocol ID: ACC#2209). Manure samples representing different stages of cow maturity (calf, milking cow, and dry cow) were collected in triplicate from each farm once each season (summer, fall, winter, spring) over 2 years. Each manure sample was obtained from three fecal pats collected from the cow pens and combined to generate a composite sample. In addition, one composite grab sample (1 L) was collected from the manure pit from each farm each sampling period that was representative of the aggregate bacterial population present in farm waste.

Environmental samples included surface water from drainage ditches or creeks and soil from fields in close proximity to where the herds were housed that may have had a manure fertilizer application. Surface water samples (1 L) were collected in triplicate from each farm during each sampling period. Composite soil samples were collected from two fields at each farm using a soil coring kit (3 cm diameter) to a depth of 10 cm. Soil samples were collected at 0 m, 45 m, and 90 m distances along a 90 m transect perpendicular to where the herds were housed and were combined to create a composite sample.

To capture the human component of the One Health continuum and complement the animal and environmental sampling from the farms, wastewater samples were collected in triplicate from two communities within the Fraser Valley in close proximity to the sampled dairy farms. One liter of composite influent (post-grit removal) and 2 L of effluent (immediately prior to release to the environment) were collected for each sample, and samples were collected at the same time points as the animal and environmental samples over the study period. All samples were transported on ice and immediately refrigerated upon arrival in the lab and processed within 24 h of collection.

### Bacterial isolation and identification

*E. coli* was recovered from manure and soil samples by re-suspending samples in buffered peptone water (BPW, Becton, Dickinson and Company; Sparks, MD, USA), plating directly onto MacConkey agar (Becton, Dickinson and Company; Mississauga, Canada) and incubating at 37°C for 18–20 h. *E. coli* was recovered from surface water and wastewater samples by filtering 100 mL at varying dilutions through a 0.45 µm membrane filter and plating onto CHROMagar *E. coli* media (Dalynn Biologicals; Calgary, Canada) and incubated at 37°C for 18–20 h.

Up to two presumptive *E. coli* colonies from each sample were purified onto Levine Eosin Methylene Blue (EMB) agar (Becton, Dickinson and Company; Mississauga, Ontario, Canada) and incubated at 37°C for 18-20 h. Presumptive *E. coli* were cultured overnight in Tryptic Soy Broth (TSB, Becton, Dickinson and Company) and preserved in 25% glycerol at −80°C.

### DNA extraction and PCR identification

DNA was extracted from the overnight cultures in TSB as described by A. Agersborg et al. (17). The identity of the presumptive *E. coli* isolates was confirmed by PCR targeting the *uspA* gene (18), with *E. coli* (ATCC 25922) used as a positive control. Each 25 µL PCR comprised of 1× OneTaq Mastermix (New England Biolabs, Ipswich, MA, USA), 0.2 µM of the forward and reverse primers (Integrated DNA Technologies, Coralville, IA, USA), and 1 µL of DNA. Amplification was carried out as follows: initial denaturation at 94°C for 5 min; 30 cycles at 94°C for 2 min, 56°C for 60 s, and 72°C for 60 s; and a final elongation step at 68°C for 5 min. A volume of 2.5 µL of 6X TriTrack DNA loading dye (Thermo Scientific, Waltham, MA, USA) was added to each PCR product, then products were run through a 2% agarose (Fisher Scientific, Hampton, NH, USA) gel containing 10% SYBR Safe (Invitrogen, Waltham, MA, USA) for 1 h at 100 V. A 100 kb plus DNA ladder (Thermo Scientific, Waltham, MA, USA) was used as a size marker. Amplicons were then visualized using the Axygen Gel Documentation System-BL and related Axygen Imaging computer software (v1.2.0.1025; Axygen, Corning, NY, USA). The identity of presumptive *E. coli* was confirmed by amplification of the *uspA* (884 bp) gene.

### Selection of a subset of isolates for further testing

A total of 1,016 *E. coli* isolates were recovered from MacConkey and Chromagar *E. coli*. Of these, a subset of 421 isolates was selected that were representative of each farming regime, sample type, and season for further analysis, including phenotypic antimicrobial susceptibility testing and short-read WGS. In all, 30 *E. coli* isolates from each sample type and farming regime were randomly selected (seed = 123) using R v4.4.3 (19). Thirty isolates from wastewater influent and 30 isolates from wastewater effluent samples were also randomly selected for sequencing. Comparisons between seasons and sample types accounted for differences in the number of isolates selected by using appropriate statistical tests with more conservative comparisons (e.g., the Bonferroni method for pairwise comparisons).

### Antimicrobial susceptibility testing

Broth microdilution testing was carried out on a subset of our *E. coli* isolates (*n* = 421) using the Sensititre system and the National Antimicrobial Resistance Monitoring System (NARMS) gram-negative plates (Panel CMV5AGNF, Thermo-Fisher, Waltham, Massachusetts, USA). Minimum inhibitory concentrations (MICs) were determined for fourteen different antimicrobials: amoxicillin-clavulanic acid combination, ampicillin, azithromycin, cefoxitin, ceftriaxone, chloramphenicol, ciprofloxacin, colistin, gentamicin, meropenem, nalidixic acid, sulfisoxazole, tetracycline, and trimethoprim-sulfamethoxazole combination. These antimicrobials represent eight different antimicrobial classes, including beta-lactams, macrolides, fluoroquinolones, sulfonamides, amphenicols, polymyxins, aminoglycosides, and tetracyclines (Table 1). The Clinical Laboratory Standards Institute (CLSI) guideline M100 was used to categorize isolates as susceptible, intermediate, or resistant for all antimicrobials tested except for azithromycin (20). No CLSI breakpoint data were available for azithromycin at the time of analysis; instead, breakpoints were determined based on the distribution of MICs and were harmonized with NARMS. Intermediate results were treated as susceptible in our analysis, and *E. coli* isolates resistant to three or more different antimicrobial classes were classified as being multi-drug resistant (MDR).

**TABLE 1:** Summary of the 14 antimicrobials that were used to assess the susceptibility of the 421 *E. coli* recovered in this study

Antimicrobial	Antimicrobial class	Health Canada (21) category^*b*^
Amoxicillin-clavulanic acid	β-lactam	I
Ampicillin	β-lactam	I^*a*^
Azithromycin	Macrolide	II
Cefoxitin	β-lactam	I^*a*^
Ceftriaxone	β-lactam	I^*a*^
Chloramphenicol	Amphenicol	III
Ciprofloxacin	Fluoroquinolone	I
Colistin	Polycationic peptide (polymyxins)	I
Gentamicin	Aminoglycoside	II
Meropenem	β-lactam	I^*a*^
Nalidixic acid	Fluoroquinolone	I
Sulfisoxazole	Sulfonamide	III
Tetracycline	Tetracycline	III
Trimethoprim-sulfamethoxazole	Diaminopyrimidine/sulfonamide	II

^*a*^ β-lactams are category I when it is a penicillin-β-lactamase inhibitor combo.

^*b*^ Category I, very high importance to human medicine; category II, high importance to human medicine; category III, medium importance to human medicine.

### Whole-genome sequencing

Genomic DNA was extracted from the 421 selected *E. coli* isolates using the Qiagen DNeasy Blood and Tissue Kits (Qiagen, Hilden, Germany) following the *Gram-Negative Bacteria Pretreatment Protocol* (p. 53) and the *Total DNA Purification from Animal Tissue Protocol* (p. 33-36) from the *DNeasy Blood and Tissue Handbook,* with several modifications as follows. Selected *E. coli* isolates were streaked onto EMB from the previously prepared 25% frozen glycerol stock and incubated at 37°C for 18–20 h. One colony from each plate was inoculated into TSB and incubated at 37°C for 18–20 h at 200 rpm. Two milliliters of liquid overnight culture was transferred to a microcentrifuge tube and centrifuged (12,000 × *g*, 5 min), resuspended in Buffer ATL, and incubated with Proteinase K (56°C, 30 min). RNase A (5 µL, 100 mg/mL) was added, followed by a 10-min incubation at room temperature. Buffer AL and 95% ethanol were added, and samples were centrifuged (16,000 × *g*, 1 min). Wash steps were performed with Buffer AW1 (16,000 × *g*, 1 min) and Buffer AW2 (5-min room temperature incubation, then centrifugation at 17,000 × *g*, 4 min). This AW2 wash was repeated. DNA was eluted twice in 100 μL of 10 mM Tris-HCl following a 5-min room temperature incubation and centrifugation (16,000 × *g*, 1 min). DNA was quantified using a ThermoScientific NanoDrop One (Product v.2.12.0; Software v.2.12.0.6). DNA extractions that did not yield a sufficient quality or purity were further cleaned up using a sodium acetate precipitation and RNase cocktail incubation (Invitrogen, Thermo Fisher Scientific, Waltham, MA, USA).

Sequencing libraries were prepared using Illumina DNA Prep Tagmentation (96 Samples, IPB) kit with Index Sets A, B, C, and D (Illumina Inc., San Diego, CA, USA), using, on average, 190 ng of DNA for each isolate. Short-read WGS was performed on an Illumina NextSeq500 (2 × 150 bp) or a NextSeq1000 (2 × 300 bp) (Illumina Inc., San Diego, CA, USA) using the NextSeq 500/550 High Output or the NextSeq 1000/2000 P2 XLEAP-SBS Reagent Kit, achieving an average sequencing depth of 60× (minimum 14× coverage). Sequencing was performed at the Molecular Technologies Laboratory (Ottawa Research and Development Center, Agriculture & Agri-Food Canada) and the Canadian Food Inspection Agency in Ottawa. Genome assembly was performed using Trimmomatic v.0.39 (22) and SPAdes v.3.15.2 (23) to filter and assemble the raw paired-end reads, respectively. Assembly statistics were then assessed using QUAST v.5.0.2 (24). Kraken2 v.2.0.9 (25) with the MiniKraken2_V1 database was used to infer and verify the taxonomic identity of each isolate. Genome characterization was performed using the VMR bioinformatics pipeline (https://github.com/grdi-amr/vmr-bioinformatics-pipeline-nf). Analysis tools were used with default settings unless noted. Serotyping was conducted using ECTyper v.1.0.0 (26). Multi-locus sequence typing (MLST) was carried out using StarAMR v.0.11.0 (27) and mlst v.2.23.0 (28), with “novel” sequence types (STs) manually verified using the PubMLST database (accessed 20 July 2025) (29). ARGs and AMR-associated point mutations were identified using StarAMR and the ResFinder v.2.5.1 (30) and Pointfinder v.4.1.1 (31) databases. Plasmids were identified using StarAMR and the PlasmidFinder database (32). The location of resistance genes as plasmid, chromosome, or both was determined by combining the ResFinder and PlasmidFinder results. Specifically, resistance genes that were identified as belonging on a contig that was identified as a plasmid were classified as being located on a plasmid. Screening for potential virulence genes was performed using ABRicate (33) and the Virulence Factors Database (VFDB, 14 January 2025) (34).

### Phylogenetic analysis

Snippy v.4.6.0 was used to generate a core single-nucleotide polymorphism (SNP) alignment by aligning each genome against a reference genome (*E. coli* AMC3019) (https://github.com/tseemann/snippy). Core alignments were processed within Gubbins 3.4 (35) to generate a maximum likelihood phylogeny using IQtree 2.4.0 (36) as the tree builder with the auto-detected best fitting model (GTR +F + ASC + G4). Treeviewer was used to visualize the respective phylogenetic trees (37). A pairwise SNP distance matrix was generated using snp-dists v.0.7.0 (https://github.com/tseemann/snp-dists). Isolate pairs with SNP differences of 100 or less were deemed closely related (38). In this context, an “isolate pair” refers to a combination of two *E. coli* isolates being compared for their genetic similarity.

### Statistical analysis and visualization

Data processing and statistical analyses were performed with R v.4.4.3 (19). The tidyverse v.2.0.0 (39) package was used for data manipulation and visualization. Clustermaps were generated using pheatmap v.1.0.13 (40), and bar plots were created using ggplot2 v.3.5.1 (41). Isolate network figures were created using ggplot2, igraph v.2.1.4 (42), and ggraph v.2.2.1 (43). Python v.3.11.9 was used to generate the upset plot and Venn diagram, using the upsetplot v.0.9.0 (44) and matplotlib-venn v.1.1.2 (matplotlib-venn · PyPI) packages, respectively.

Genetic element data from the VMR pipeline were statistically analyzed using R. Due to overdispersion and large variance in the number of ARGs and virulence genes present in isolates, a negative binomial regression model was used to evaluate the effect of season and sample source on the number of these genetic factors in isolates. A Poisson regression was performed to evaluate the effect of season and sample source on the number of plasmids in each isolate due to lower variance and lack of overdispersion. The MASS package v.7.3-65 (45) was used to perform initial likelihood ratio tests comparing the respective model with and without season or sample source as a predictor of the number of genetic determinants per isolate. Post hoc pairwise comparisons (Bonferroni-adjusted) were conducted using the emmeans package v.1.11.1 (46) for all genetic elements (ARGs, virulence genes, and plasmids).

## RESULTS

### Source-driven and seasonal variation in *E. coli*

A total of 1,016 *E. coli* isolates were recovered from farm, wastewater, and environmental samples (Table 2), and 421 isolates were subjected to WGS. Of the samples collected, calf feces had the highest concentration of *E. coli* (1.1 × 10^8^ CFU/g) while environmental samples had the lowest (7.94 × 10^2^ CFU/g of soil and 1.28 × 10^3^ CFU/100 mL surface water) (*P* < 0.0001, emmeans). Some significant differences were also found between seasons for dry cow feces, manure pit, manured soil, surface water, and wastewater effluent samples (Table S1). Among these samples, different seasons had significantly higher concentrations of *E. coli*, suggesting that season influences *E. coli* concentration in a non-uniform manner amongst these sample sources. No significant differences between seasons were found for calf feces, milking cow feces, or wastewater influent samples.

**TABLE 2:** Summary of total number of *E. coli* isolates collected for each sample source in each season and the subset selected for antimicrobial susceptibility testing and WGS

	Summer	Fall	Winter	Spring	Total
Total *E. coli* isolates*^a^*					
Calf feces	18	22	31	38	109
Milking cow feces	48	54	53	64	219
Dry cow feces	47	51	57	52	207
Manure pit	38	28	42	44	152
Manured soil	7	35	15	19	76
Surface water	36	52	41	50	179
Wastewater influent	1	6	13	10	30
Wastewater effluent	2	16	12	14	44
Total	197	264	264	291	1,016
Subset of isolates^*b*^					
Calf feces	10	10	20	20	60
Milking cow feces	12	12	15	21	60
Dry cow feces	12	12	16	20	60
Manure pit	12	8	21	19	60
Manured soil	7	34	9	11	61
Surface water	12	12	17	19	60
Wastewater influent	1	6	13	10	30
Wastewater effluent	2	6	11	11	30
Total	68	100	122	131	421

^*a*^ Total number of *E. coli* isolates collected for each sample source in each season.

^*b*^ Subset of *E. coli* isolates selected for antimicrobial susceptibility testing and WGS.

### Limited AMR with source-specific MDR in *E. coli*

**Fig 1:**
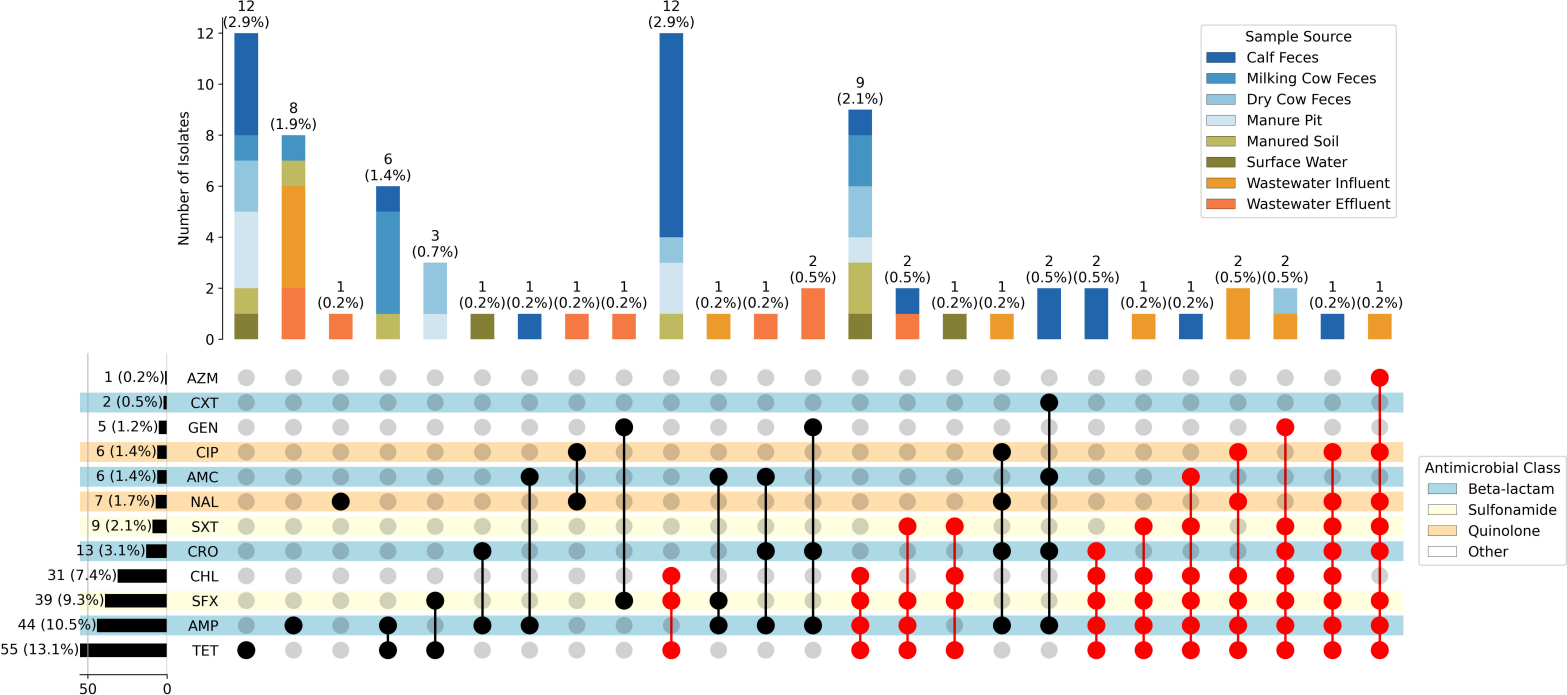
Summary of resistance profiles of *E. coli* isolates (*n* = 421) collected from animal, environmental, and human wastewater sources. Resistance profiles are represented by the bottom plot, where each dot indicates a resistant phenotype. Red dots indicate a MDR phenotype. Antimicrobials are represented by abbreviations as follows: azithromycin (AZM), cefoxitin (CXT), gentamicin (GEN), ciprofloxacin (CIP), amoxicillin/clavulanic acid (AMC), nalidixic acid (NAL), trimethoprim/sulfamethoxazole (SXT), ceftriaxone (CRO), chloramphenicol (CHL), sulfisoxazole (SFX), ampicillin (AMP), and tetracycline (TET).

Antimicrobial susceptibility testing revealed that 82.19% (346/421) of the *E. coli* isolates were susceptible to all fourteen antimicrobials tested. None of the isolates were resistant to colistin or meropenem (Fig. 1). Of the remaining 75 *E. coli* isolates, resistance to tetracycline and ampicillin was the most common, occurring in 13.06% (55/421) and 10.45% (44/421) of isolates, respectively. The majority of the 75 AMR *E. coli* isolates (54/75; 72.00%) were resistant to more than one antimicrobial, and almost half of these isolates (34/75; 45.33%) were classified as MDR. In all, 25 different resistance profiles were present, 11 of which were MDR profiles (Fig. 1). Among MDR profiles, resistance to chloramphenicol, sulfisoxazole, and tetracycline was the most common, occurring in 16.00% (12/75) of resistant isolates (Fig. 1). Resistance to ampicillin, sulfisoxazole, tetracycline, and trimethoprim-sulfamethoxazole was the second most common resistance profile, found in nine isolates, and was found in all sample sources except for wastewater influent and effluent (Fig. 1). No significant differences in the occurrence of resistant phenotypes or MDR were found between seasons (*P >* 0.05, Fisher’s exact test). Overall, calf fecal samples and wastewater influent samples had a higher occurrence of resistant phenotypes and MDR than other sample types, and calf fecal samples had a significantly higher occurrence of MDR phenotypes compared to all other sample sources except for wastewater influent (*P >* 0.05, Fisher’s exact test, Bonferroni correction).

### Serotype and ST variation across sample sources

**Fig 2:**
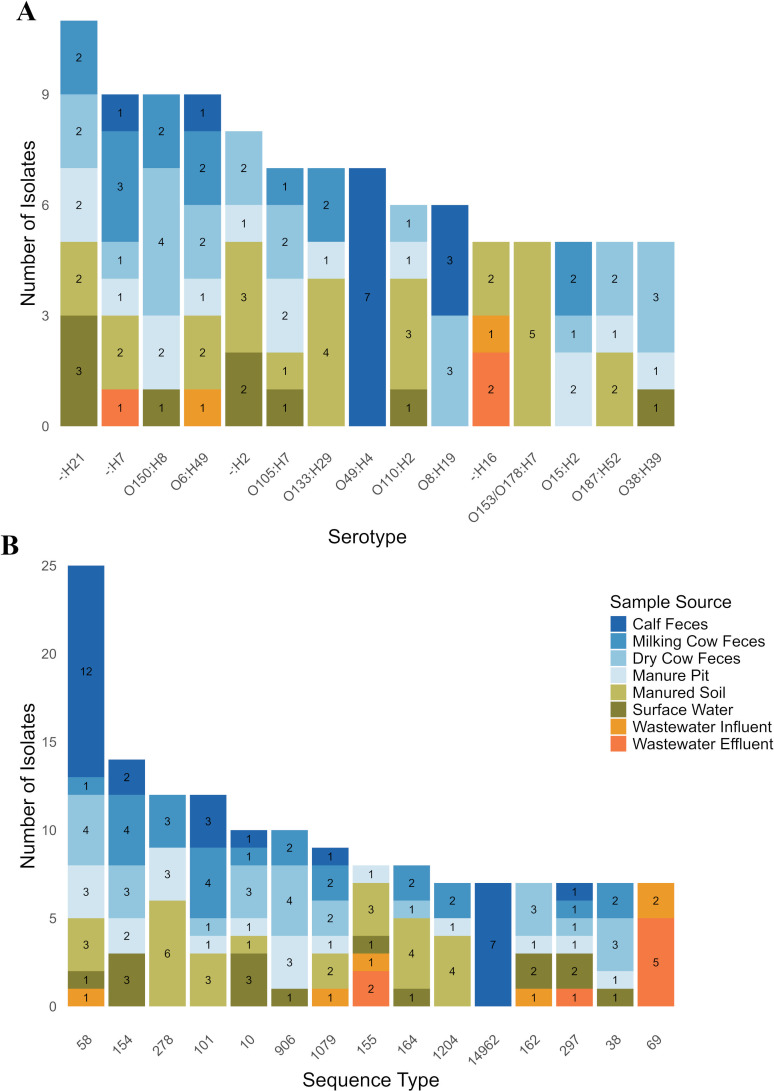
Fifteen most common serotypes (**A**) and sequence types (**B**) found among *E. coli* isolates (*n* = 421) recovered from dairy farms, environmental, and human wastewater samples. If no O-typeable antigen was present, the O antigen is represented as a dash.

Among the 421 sequenced *E. coli* isolates, 211 different serotypes were found (Fig. 2; Table S1). Of these, 59 lacked a typeable O antigen under default settings, and two lacked both typeable O and H antigens. The most common serotype was H21 with an untypeable O antigen (*n* = 11) (Fig. 2A). Among the 15 most common serotypes, there was representation from at least 2 of the 3 One Health domains for 11 of the 15 serotypes, with animal and environmental samples often sharing similar serotypes (Fig. 2A). Serotype O49:H4 was only found in calf fecal samples (*n* = 7), serotype O8:H19 was only found in calf and dry cow samples (*n* = 3 for calves and 3 for dry cows), and serotype O153/O178:H7 was only found in manured soil samples (*n* = 5) (Fig. 2A).

Among the 421 *E. coli* isolates, we identified 174 different STs, including 8 novel STs (ST18490-18497) (Fig. 2B; Table S2). The 15 most common STs accounted for 150 isolates, with 13 of these detected in at least 2 of the 3 One Health domains. ST58 was the most common (*n* = 25/421; 5.94%) and was observed across the entire One Health continuum, while ST14962 (*n* = 7) and ST69 (*n* = 7) were found exclusively in calf fecal samples and wastewater samples, respectively (Fig. 2B).

### Diverse ARGs with source-dependent distribution in *E. coli*

**Fig 3:**
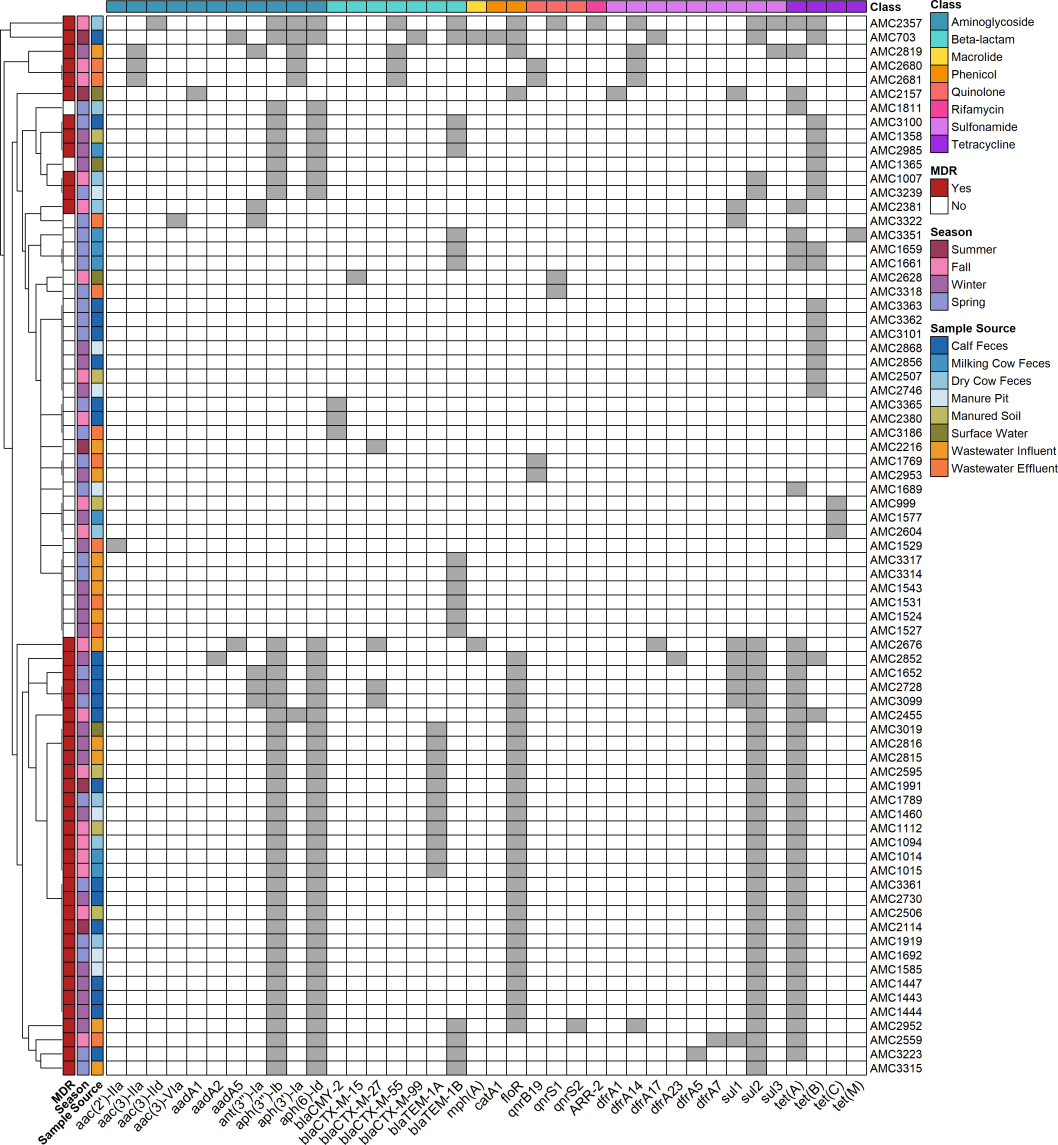
Clustermap showing the presence (gray boxes) of different ARGs (x-axis) in *E. coli* isolates (*n* = 75) recovered from manure, environmental, and human wastewater samples. Sample source and season are indicated by colored boxes along the left y-axis. MDR isolates, defined as those carrying resistance to three or more antimicrobial classes, are highlighted in red on the left y-axis. The antimicrobial class associated with each gene is represented by colored bars on the top x-axis. Isolates with similar resistance gene profiles are grouped hierarchically, as shown by the dendrogram on the left.

**Fig 4:**
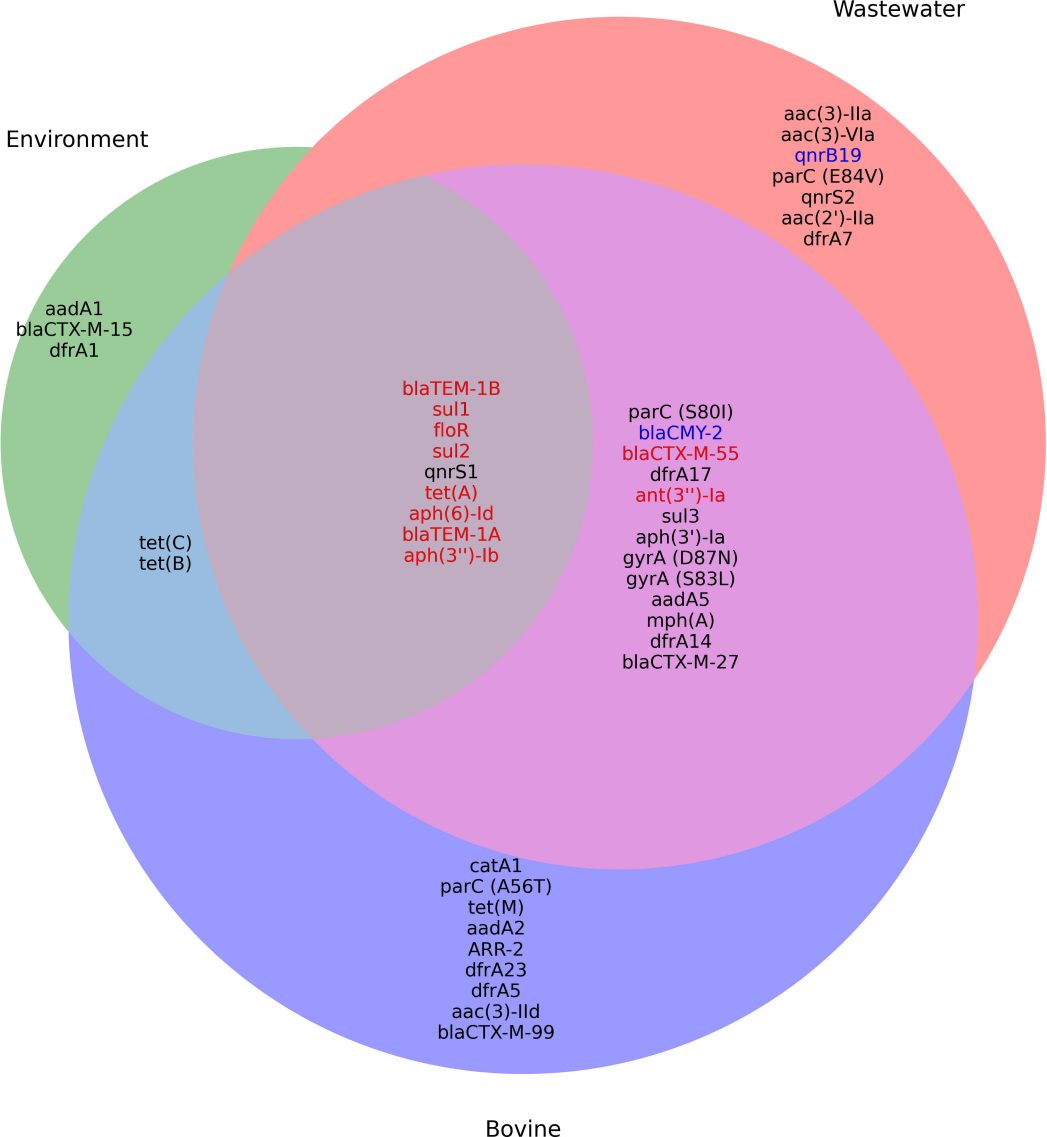
Venn diagram of shared ARGs found in *E. coli* isolates recovered across the One Health continuum. Black text indicates chromosomally encoded genes, blue indicates plasmid, and red indicates both (chromosomally and plasmid).

Of the 421 *E. coli* isolates sequenced, 38 different ARGs conferring resistance to eight different antimicrobial classes were found, with 17.8% (75/421) of isolates possessing at least one ARG (Fig. 3). Of the ARGs detected, *aph*(3″)-Ib and *aph*(6)-Id genes which confer resistance to aminoglycosides were the most common (40/421), followed by *tet*(A) (39/421). Point mutations conferring AMR were found in seven of the 421 isolates and included two mutations in the *gyr*A gene and three in subunit A of the topoisomerase IV (*par*C) gene. Season did not have a significant influence on the number of ARGs found in isolates (likelihood ratio test: chi-squared test (df = 3) = 1.94, *P =* 0.586). In contrast, the sample source was found to influence the number of ARGs detected per isolate (likelihood ratio test: chi-squared test [df = 7] = 17.96, *P =* 0.012). Post hoc pairwise comparisons (Bonferroni-adjusted) did not yield statistically significant differences between different sample sources after correction. Of the 38 ARGs detected, nine were found in all three One Health domains, and 13 were found shared between the animal and human domains (Fig. 4). Of the ARGs found in all three domains, all except one (*gnrS1*) were both chromosomally and plasmid encoded.

Of the 75 isolates that had at least one ARG, 57.33% (43/75) were classified as MDR. Eleven different MDR profiles were identified, with the most common MDR profiles including resistance to aminoglycosides, phenicols, sulfonamides, and tetracyclines, with or without resistance to beta-lactams (*n* = 14 for both). MDR genotypes occurred in all sample types representing all aspects of the One Health continuum, but more MDR genotypes were detected from *E. coli* isolated in winter (*n* = 15) compared to summer (*n* = 4). MDR genotypes also occurred most frequently in calf fecal samples (*n* = 15) and least frequently in surface water samples (*n* = 2).

### High plasmid prevalence with source- and season-dependent variation

**Fig 5:**
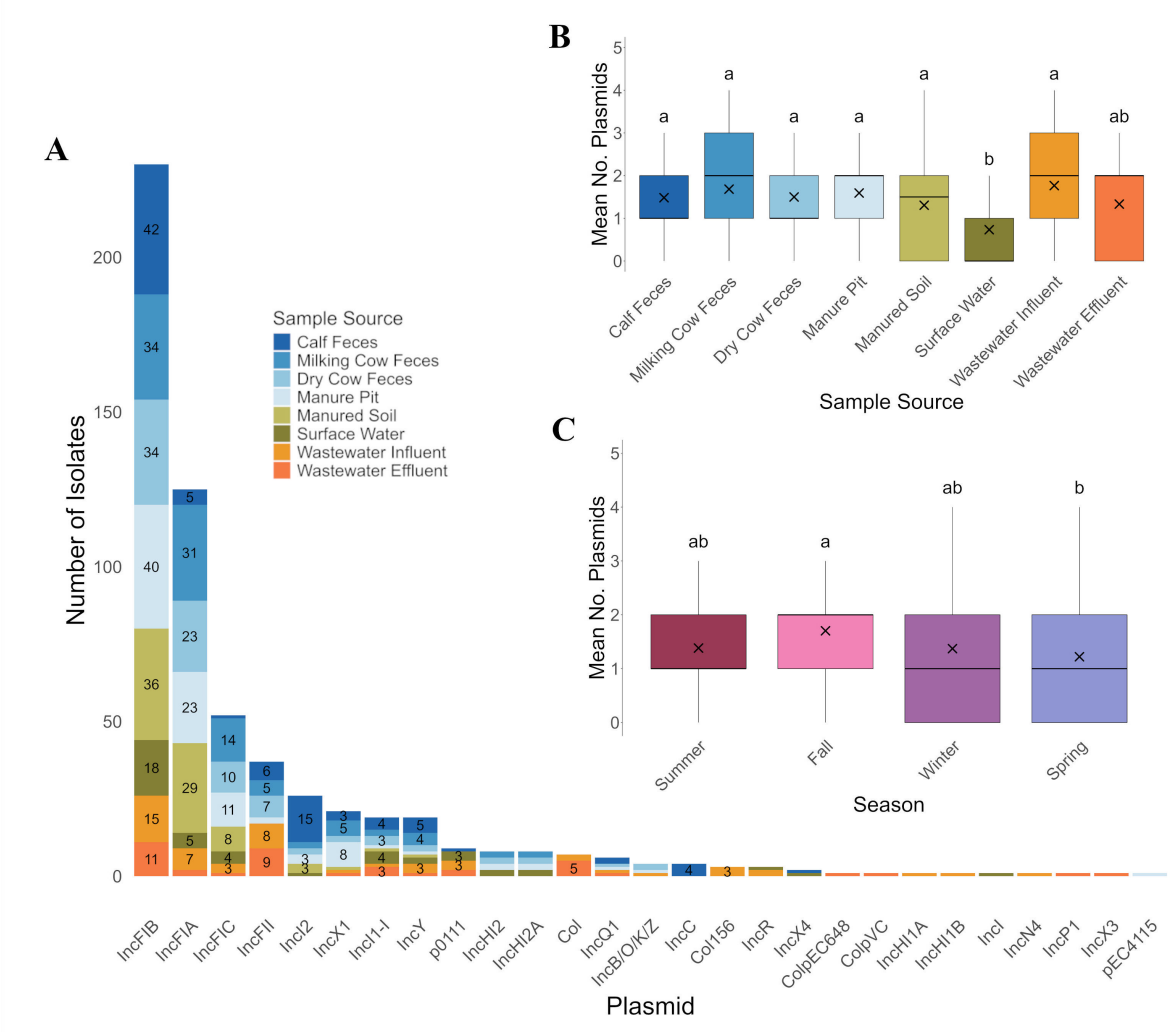
Distribution of plasmid replicon types in *E. coli* by sample source (**A**) and the average number of plasmids by sample source (**B**) and season (**C**). Numbers represent the total number of isolates from each sample source that possessed at least one replicon of the plasmid. Different letters indicate significant differences according to emmeans pairwise comparisons (*P* < 0.05).

Plasmids were found in 305 of the 421 *E. coli* isolates (Fig. 5A) and included 27 different replicons. The majority of *E. coli* isolates had only 1–2 plasmids (*n* = 242), 61 isolates had 3–4 plasmids, and a handful of isolates had as many as five plasmids (*n* = 2). The *IncFIB* plasmid was the most common (*n* = 230), followed by *IncFIA* (*n* = 125) and *IncFIC* (*n* = 52) (Table S3). Over half of the isolates (254/421) had at least one of these replicon types, and 33 isolates had all three. Nine of the plasmids were only observed in a single isolate, with seven of these occurring in isolates recovered from human wastewater. The number of plasmids per isolate differed significantly across sample types (likelihood ratio test: chi-squared test [df = 7] = 87.76, *P >* 0.0001), with fewer plasmids found in isolates recovered from surface water (Fig. 5B). Season also influenced plasmid occurrence, with isolates recovered in spring having significantly lower numbers of plasmids than isolates obtained in the fall (*P* = 0.015) (Fig. 5C).

### Extensive virulence gene diversity with source-dependent toxin gene patterns

**Fig 6:**
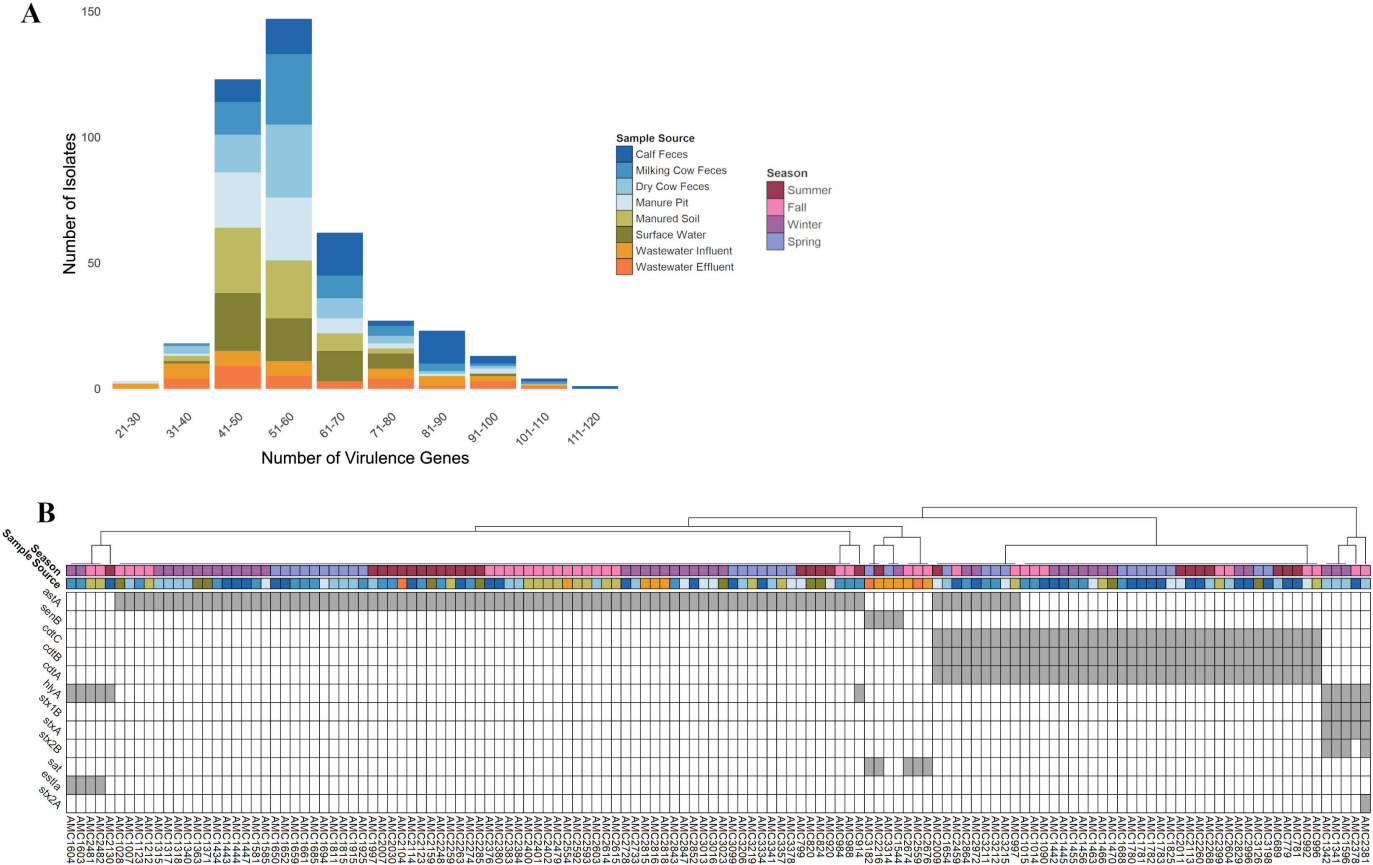
Summary of the number of virulence genes found in *E. coli* isolates (*n* = 421) according to sample type (**A**) and (**B**) clustermap showing the presence/absence of toxin genes detected among isolates (*n* = 134). Toxin genes are depicted on the left y-axis, with a gray box indicating the gene was present. Each “AMC” number on the bottom x-axis represents a single isolate.

In all, 241 different virulence genes were identified among the 421 *E. coli* isolates (23–111 genes per isolate) (Fig. 6A; Table S4). Thirty-nine of the 241 virulence factors were part of the “core virulome” (present in ≥90% of isolates). In contrast, 158 virulence factors were detected in less than 10% of isolates. Twelve different toxin genes were identified (Table S4), occurring in 31.83% (134/421) of isolates, with isolates carrying as many as five different toxin genes (Fig. 6B). The *astA* toxin gene was the most common (*n* = 86), followed by *cdtA, cdtB,* and *cdtC* (*n* = 40). Diverse representation of the occurrence of toxin genes was observed across sample sources and seasons. Variants of the *stx* gene (*stxA*, *stx1B*, *stx2B*, and *stx2A)* were only detected in isolates recovered from animal samples (dry cow feces and calf feces). Isolates recovered from calf feces had the highest number of toxin genes present (*n* = 67), while isolates recovered from wastewater influent and effluent had the least (*n* = 10 and 4, respectively).

Season was not found to have a significant influence on the number of toxin genes found in isolates (*P* = 0.456). In contrast, the number of toxin genes detected per isolate differed significantly across the different sample types (likelihood ratio test: chi-squared test [df = 7] = 35.37, *P* > 0.00001). The number of toxin genes was significantly lower in surface water compared to calf feces (*P* = 0.0006), dry cow feces (*P* = 0.0550), and milking cow feces (*P* = 0.0288). Calf feces had significantly more toxin genes than wastewater effluent (*P* = 0.0072).

### Genomic relatedness shows source-dependent clustering of *E. coli*

**Fig 7:**
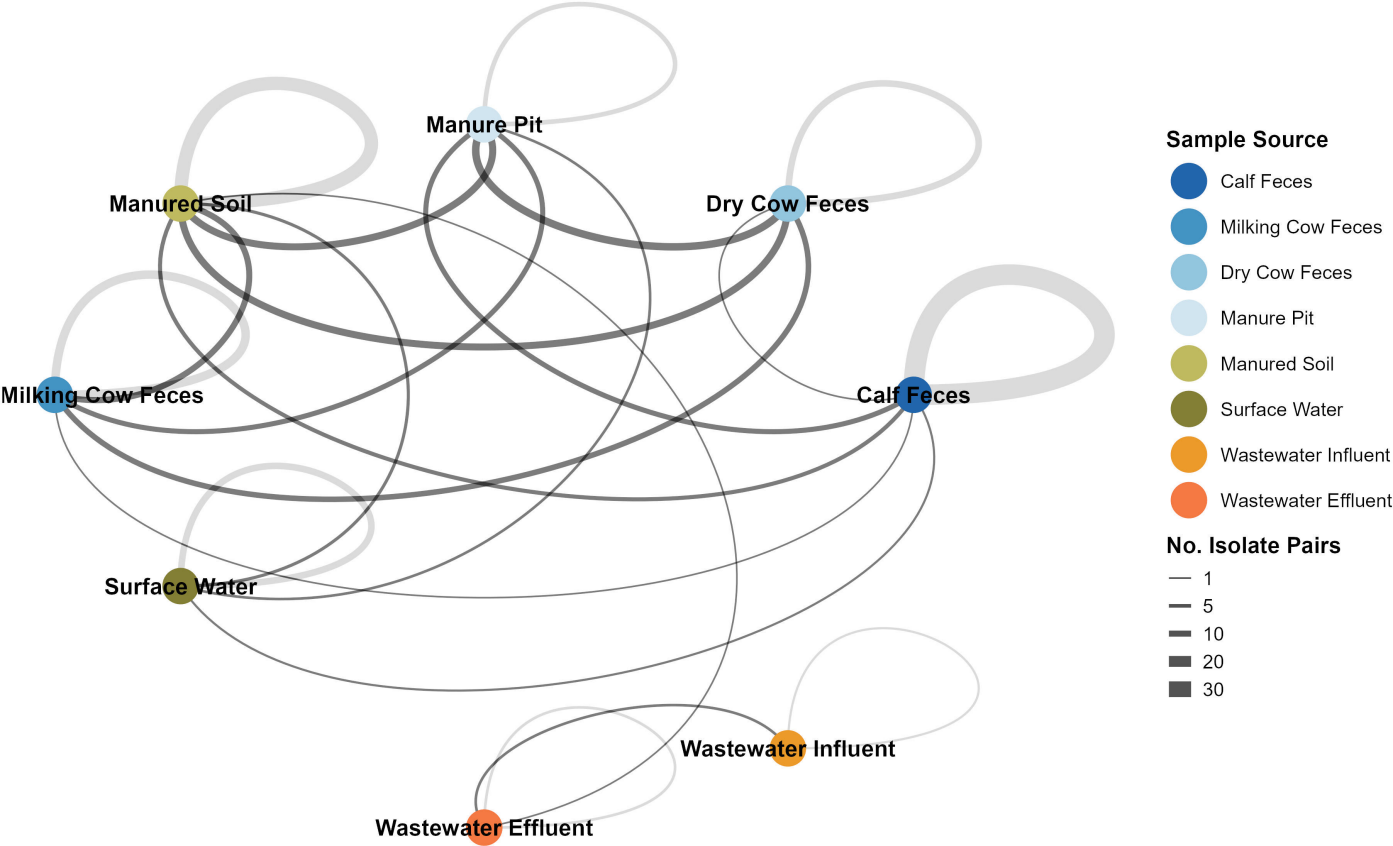
Network visualization of closely related (≤100 SNPs) *E. coli* isolates based on pairwise SNP differences. Each node represents a distinct sample source (manure, environmental, and wastewater) from which *E. coli* isolates were recovered seasonally over a 2-year period. Lines between nodes indicate connections between closely related isolates from each respective sample source, with line thickness proportional to the number of isolate pairs deemed closely related. Self-loops (gray arcs) represent closely related isolates of the same sample source.

Pairwise SNP differences among our isolates ranged from 0 to 126,160 SNPs (Table S5; Fig. S1). Among the >88,000 SNP pairwise comparisons, 207 isolate pairs (representing less than 0.25% of all comparisons) exhibited 100 or fewer SNP differences and were considered closely related. These closely related pairs predominantly (114/207; 55.07%) involved isolates from the same sample source (Fig. 7). Approximately 25% (46/207) of the closely related pairs were from different One Health domains. The remaining 47 isolate pairs were between isolates from different sources but within the same One Health domain (e.g., dry cow feces and manure pit). In total, 213 of the 421 *E. coli* isolates (50.59%) had a close relationship with at least one other isolate, and 114 (27.08%) of all isolates had at least one close relationship with an isolate from a different sample source. Notably, wastewater influent and effluent isolates were almost exclusively closely related to other wastewater isolates, with only one close genetic linkage (92 SNP difference) identified between wastewater effluent and an isolate from manured soil.

### Genomic features of beta-lactam resistant isolates

**Fig 8:**
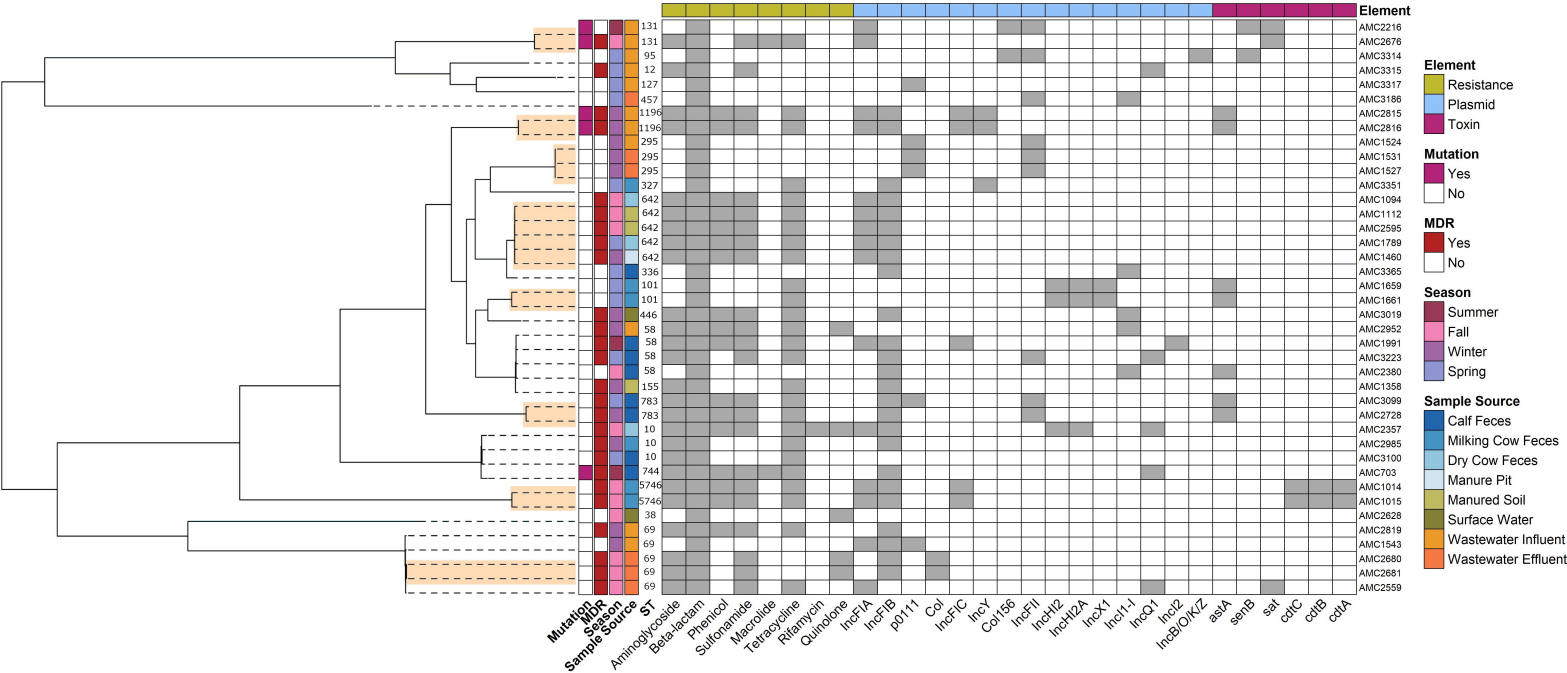
Clustermap summarizing the genomic features of 40 *E. coli* isolates that possessed a beta-lactam resistance gene (*bla*_CMY-2_, *bla*_CTX-M-15_, *bla*_CTX-M-27_, *bla*_CTX-M-55_, *bla*_CTX-M-99_, *bla*_TEM-1A_, or *bla*_TEM-1B_). Gray boxes indicate the corresponding gene or genetic element was present. Genetic elements include plasmids, toxin genes, and ARGs, which are summarized by antimicrobial class. The “Mutation” column indicates the presence of point mutations associated with resistance. Isolates with ARGs conferring resistance to three or more antimicrobial classes are classified as MDR as shown in the second column from the left. ST is listed on the left y-axis. Isolates are clustered by genomic relatedness based on SNP differences, with dendrogram branch lengths proportional to relatedness. Closely related isolates (≤100 SNP differences) are highlighted in the tree on the left in orange.

Among the 421 *E. coli* isolates, 40 isolates, representing all sample types, had at least one gene conferring beta-lactam resistance (Fig. 8). Isolates harboring a beta-lactam ARG were often recovered from wastewater influent (*n* = 11), while only a single manure pit isolate possessed a beta-lactam ARG. Representation of STs and serotypes within these isolates was diverse, with 20 different STs identified, with ST642 and ST69 the most common. ST69 was recovered exclusively from human wastewater samples. ST642 had the same toxin genes, plasmid replicons, and resistance profiles across all five isolates, while ST69 showed some variation in these elements between isolates. Over half of the 40 isolates (65%; 26/40) were MDR. Six different toxin genes were found among the 40 beta-lactam-resistant isolates, including *astA* (*n* = 7), *cdtA* (*n* = 2), *cdtC* (*n* =2), *cdtB* (*n* = 2), *sat* (*n* = 3), and *senB* (*n* = 3). Fifteen plasmid replicons were also found among the 40 isolates, with all isolates harboring at least one plasmid. *IncFIB* was the most common replicon type (*n* = 25), followed by *IncFIA* (*n* =15).

Phylogenomic comparison of the 40 beta-lactam-resistant *E. coli* isolates (0–79,952 SNPs) highlighted that wastewater isolates tended to cluster independently of dairy and environmental isolates (Fig. 8). Of the 780 SNP pairwise combinations, 19 isolate pairs exhibited fewer than 100 SNP differences and were considered closely related. Four of these closely related pairs were between dry cow isolates and manured soil isolates. All other closely related pairs occurred in two or fewer instances, with no close genetic relationships detected among surface water isolates. Isolates recovered from milking cow feces and calf feces showed close relationships only with isolates from the same sample source and did not have any close relationships detected with other sample sources. No close relationships were detected between wastewater isolates and isolates from other sources. With the exception of one isolate (AMC2952), wastewater isolates generally clustered separately from isolates recovered from animal or environmental sources, and isolates of the same ST tended to cluster together (Fig. 8).

## DISCUSSION

We assessed the occurrence of AMR *E. coli* and its genetic determinants across all three One Health domains to evaluate the risk of cross-domain transmission. The most common serotype was ONT:H21. Notably, the O157:H7 serotype commonly associated with Shiga toxin-producing *E. coli* (STEC) and a major cause of severe illness in humans (47) was absent in our collection of isolates. Among the top 6 non-O157 STEC serogroups (O26, O45, O103, O111, O121, and O145) most frequently associated with human disease (48, 49), only 10 of our isolates carried one of these antigens. The ONT:H25 serotype, commonly associated with zoonotic pathogen transmission and often found in dairy cattle (50), was also absent. This scarcity of high-risk pathogenic serotypes, combined with the low abundance of high-risk toxin genes (*cdtABC*, *estIa*, *sat*, *stx*), suggests that the isolates in this study may have relatively low pathogenic potential (51).

Among the 15 most common STs, three (ST10, ST38, and ST69) were among the most frequently reported high-risk pandemic lineages commonly associated with human infections. High-risk pandemic lineages are often globally distributed, have enhanced pathogenicity, and can colonize and persist in hosts, causing severe or recurrent infections that can be transmitted to other hosts. Pandemic lineages also often contain ARGs and other genetic determinants of AMR and therefore play a significant role in the dissemination of AMR (52, 53). ST10, ST38, and ST69 are generally associated with extraintestinal pathogenic *E. coli* (ExPEC) strains (51) and are commonly associated with healthcare and community-associated infections (54). A total of 24 isolates in our collection belonged to these three STs. Isolates from animal and environmental sources belonged to ST10 and ST38, while ST69 was exclusively found in human wastewater. The absence of ST10 and ST38 in wastewater, combined with the exclusive presence of ST69 in this source, suggests that ST69 likely originates from the human population, while ST10 and ST38 likely originate from animal or environmental reservoirs. Additionally, two isolates belonged to ST131, another pandemic lineage commonly associated with human disease and known for its ability to cross One Health boundaries (55). Similar to ST69, ST131 was only recovered from human wastewater, indicating that while high-risk pandemic lineages can exist in different One Health domains, their transmission across all three elements may be limited, with some lineages restricted to specific niches.

The most common ST among our *E. coli* isolates was ST58, which was found across all three One Health domains. Although ST58 typically belongs to the non-pathogenic commensal phylogroup (56, 57), ST58 strains harboring the ColV plasmid have been associated with increased pathogenicity and ARG acquisition (57). Fortunately, the ColV plasmid was absent from all 25 ST58 isolates in this study, suggesting they are likely non-pathogenic commensals. Nevertheless, ST58 exhibits other traits associated with ExPEC isolates (56) and has a high level of phylogenomic relatedness to other high-risk pandemic lineages such as ST131, ST10, and ST38 (53). Consequently, its widespread detection across all three One Health elements, coupled with its status as the most prevalent ST, raises potential concern. Furthermore, among the 15 most common STs, thirteen were detected in at least two of the three One Health domains, with only two STs unique to a single domain—ST14962 was only detected in calf feces, whereas ST69 was only detected in wastewater. STs were frequently shared between animal and environmental sources, whereas overlaps with wastewater sources were less, suggesting a closer interaction between the animal and environmental domains compared to the human domain.

Seventy-five of the 421 *E. coli* had at least one ARG detected, and the same number displayed a resistant phenotype. Although the majority of isolates (82.19%; 346/421) were susceptible to all tested antimicrobials and lacked detectable ARGs, resistant phenotypes and ARGs were identified across all sample sources. Notably, MDR phenotypes and genotypes were present in every sample source, indicating that MDR is not confined to a single domain of the One Health continuum but can emerge throughout its various components. Animal sources had a higher occurrence of ARGs, resistant phenotypes, and MDR phenotypes compared to environmental or human wastewater samples. This higher occurrence of resistance traits in animal sources could be attributed to antimicrobial treatment in cows (e.g., blanket dry cow therapy) and management practices such as group-housing and the feeding of waste milk from cows receiving antimicrobial treatment to calves (58–60). However, while these results could be the result of the above and support the notion that large livestock animals can act as a reservoir and possible transmission source for AMR *E. coli* (3, 61), this pattern could also be due to chance, as few isolates were classified as MDR. Furthermore, the number of ARGs present in an isolate seemed to be statistically unrelated to the sample source, suggesting that ARGs and MDR phenotypes are randomly distributed across the One Health continuum, as observed by P. Leekitcharoenphon et al. (1).

In contrast, the number of plasmids and toxin genes present in an isolate appeared to be somewhat related to the sample source. Surface water isolates harbored significantly fewer plasmids compared to all other sample sources except for wastewater effluent and significantly fewer toxin genes compared to cow fecal samples (calf, dry cow, and milking cow). Additionally, wastewater effluent had significantly fewer toxin genes compared to calf feces. The lower occurrence of genetic determinants such as plasmids and toxin genes in surface water suggests that these elements and the *E. coli* strains carrying them may not be transmitted from animal sources to surface water or may not persist once introduced. Previous research has shown that carrying plasmids, toxin genes, and other AMR determinants imposes a fitness cost (62, 63). This cost may be amplified or ultimately exploited in aquatic environments such as surface water and wastewater effluent, where nutrient availability, microbial community composition, and environmental conditions differ from those in manure or wastewater influent (64), which are dominated by enteric sources. Consequently, isolates in these aquatic environments may lose plasmids and toxin genes to reduce fitness costs and improve survival.

While a large number of virulence genes were detected among our isolates, only a small number were toxin genes. Of the 12 different toxin genes detected, 4 were *stx* gene variants responsible for Shiga-toxin production and were found in isolates from dry cow feces and calf feces. Similarly, the *hlyA* gene, encoding a hemolysin that causes host cell lysis, was detected exclusively in fecal and environmental isolates. Notably, all five of the isolates with a *stx* gene also carried the *hlyA* gene, suggesting these isolates were highly virulent and pathogenic. Although these toxin genes were present in a few isolates, they are often associated with more severe disease outcomes (65). The detection of *stx* and *hlyA* genes in dairy cows emphasizes the need for interventions to prevent the dissemination of *E. coli* and genetic determinants such as toxin genes across the One Health continuum. The near-exclusive detection of *stx* genes in dry cows suggests this life stage may be particularly susceptible to harboring Shiga-toxin production genes, indicating a critical target for intervention strategies. During the drying-off period, dry cows are typically treated with antimicrobials to prevent mastitis, which may select for pathogenic or resistant strains of *E. coli* harboring *stx* genes. In contrast, *hlyA* was detected across all cow life stages (calf, milking cow, dry cow) as well as in soil samples, highlighting the ubiquitous nature of *E. coli* and the genes it harbors. These findings underscore the importance of applying a One Health approach to mitigate the spread of *E. coli* and AMR.

Overall, genomic analysis of the 421 selected *E. coli* isolates suggests that AMR and its genetic determinants are uncommon across all domains of the One Health continuum. Animal sources generally exhibited a higher occurrence of genetic determinants such as plasmids, point mutations, and virulence genes, highlighting their potential role as reservoirs. Nevertheless, the presence of genetic determinants alone does not confirm transmission events. This phylogenomic analysis revealed that our collection of 421 *E. coli* isolates is highly diverse. This relatively low proportion of close relationships between different domains suggests that while cross-domain transmission is likely occurring, it is infrequent and restricted to the farm. Wastewater influent and effluent isolates were generally more closely related to other wastewater isolates; however, one close relationship was observed between a wastewater effluent isolate and a manured soil isolate. These isolates originated from the same city, and given that wastewater effluent and manured soil typically have minimal direct interaction, this finding may indicate indirect transmission. Although evidence of cross-domain spread is concerning, the concurrent low detection of resistant genotypes and phenotypes is encouraging. Proactively implementing mitigation strategies while AMR levels remain relatively low could help reduce the risk of widespread dissemination of AMR *E. coli*.

Beta-lactam resistance was detected in 40 of the 421 *E. coli* isolates (9.50%). These isolates carried at least one beta-lactamase gene (*bla*_CMY-2_, *bla*_CTX-M-15_, *bla*_CTX-M-27_, *bla*_CTX-M-55_, *bla*_CTX-M-99_, *bla*_TEM-1A_, or *bla*_TEM-1B_), with *bla*_TEM-1B_ being the most common. Since beta-lactams are classified as Category I antimicrobials critically important for human medicine (21), the presence of these genes is particularly concerning due to the potential reduction in treatment options for patients infected with one of these bacteria (2). Among these 40 beta-lactam resistant isolates, 18 were recovered from animal sources, 17 from human wastewater, and 5 from environmental sources, underscoring the occurrence of beta-lactam resistance across all One Health domains. However, although beta-lactam-resistant *E. coli* was detected across all One Health domains, phylogenomic analysis revealed that many of the closely related isolates originated from similar sample sources. For example, wastewater isolates tended to cluster together, as did animal isolates, with occasional overlap between manured soil and animal sources. We identified two beta-lactam-resistant isolates from surface water, among the 60 isolates that underwent WGS; however, these isolates were not closely (>100 SNPs) related to isolates from other sources. Of the 40 beta-lactam-resistant isolates, only 17 isolate pairs had fewer than 100 SNP differences and were considered closely related. Of these 17 isolate pairs, four of them were between dry cow and manured soil isolates, while all other pairs occurred two times or less. While this is a low number, the higher proportion of dry cow-manured soil isolate pairs may suggest potential transmission of beta-lactam-resistant *E. coli* from dry cows to the natural environment. Overall, these findings suggest that while there is genomic overlap, most close genetic relationships occur within sample sources, rather than between, indicating low levels of transmission of beta-lactam-resistant *E. coli* between different One Health domains and suggesting that beta-lactam resistance may be a niche adaptation. Furthermore, dry cows are typically treated with antimicrobials (e.g., Cefa-Dri [Cephapirin benzathine]), which may select for beta-lactam resistant *E. coli*, and three of the six farms allowed their dry cows to graze on pastureland, possibly explaining the close relatedness between isolates recovered from dry cows and manured soil and identifying a possible transmission route.

Although widespread transmission of beta-lactam-resistant *E. coli* was not observed, several isolates carried extended-spectrum beta-lactamase (ESBL) genes, which are particularly concerning due to their broader range of resistance to beta-lactam antimicrobials and higher affinity for MDR compared to standard beta-lactamases (66). ESBL-producing *E. coli* are also often associated with a higher risk of MDR (67, 68), further limiting treatment options for infected patients. Furthermore, the World Health Organization (WHO) has classified ESBL-producing *Enterobacteriacea*e as a priority pathogen for new antibiotic development due to their resistance to a broad range of antimicrobials critical for human health (69), making their detection significant. Of the beta-lactam ARGs detected, *bla*_CTX-M-15_, *bla*_CTX-M-27_, *bla*_CTX-M-55_, and *bla*_CTX-M-99_ are classified as ESBLs, while *bla*_CMY-2_, *bla*_TEM-1A_, and *bla*_TEM-1B_ are not ESBL genes but still confer resistance to beta-lactam antimicrobials. Only 10 of the 40 beta-lactam-resistant isolates harbored a true ESBL gene. These ESBL isolates were detected across all One Health domains. Specifically, *bla*_CTX-M-55_ and *bla*_CTX-M-27_ were found in both wastewater and cattle isolates, with isolates from the same source showing close genomic relatedness. The *bla*_CTX-M-15_ and *bla*_CTX-M-99_ genes were only detected once each, in surface water and calf feces, respectively. Although ESBL-producing *E. coli* represented less than 3% of the total isolates, their presence across all One Health domains highlights the potential for transmission. Proactive improvement of current AMR mitigation strategies and implementation of novel measures is critical to containing or eliminating high-priority pathogens, such as ESBL-producing *E. coli.*

While this study was relatively comprehensive and examined all three One Health domains over a 2-year time period, there are some limitations. The sampling design included a higher representation of the animal domain compared to the environmental and human domains. This study also examined the general *E. coli* population rather than specifically selecting resistant *E. coli* strains. While this provides valuable insight into the occurrence of AMR in the general population, targeted studies focusing on AMR *E. coli*, particularly ESBL-producing strains, would provide insight into the prevalence and transmission of clinically relevant resistance genes and the dissemination of priority pathogens such as ESBL-producing *E. coli.*

In conclusion, AMR *E. coli* was detected across all three One Health domains, though overall levels of resistance and virulence were relatively low. The multi-domain presence of high-risk lineages and ESBL-producing strains indicates that cross-domain transmission is possible, even if currently limited, highlighting the importance of proactive One Health surveillance and intervention. Our findings also underscore the value of including environmental sources alongside human and animal populations, as this can reveal reservoirs and early transmission pathways that would otherwise remain undetected. Targeted management practices, such as interventions during critical cow life stages and enhanced wastewater and farm biosecurity measures, combined with expanded genomic surveillance, offer a strategic approach to preventing the emergence and dissemination of AMR before it becomes widespread.

## Data Availability

The sequencing data have been deposited in the NCBI database under BioProject PRJNA1328809.

## SUPPLEMENTAL MATERIAL

Figure S1 aem.01905-25-s0001.tif Phylogenetic tree of *E. coli* isolates obtained in this study.

Supplemental legend aem.01905-25-s0002.docx Legend for Fig. S1.

Table S1 aem.01905-25-s0003.xlsx *E. coli* concentration by sample type and season.

Table S2 aem.01905-25-s0004.xlsx Summary of all STs by sample type.

Table S3 aem.01905-25-s0005.xlsx Summary of plasmids found.

Table S4 aem.01905-25-s0006.xlsx Summary of toxin genes found.

Table S5 aem.01905-25-s0007.xlsx Summary of isolate pairs <100 SNPs.

ASM does not own the copyrights to Supplemental Material that may be linked to, or accessed through, an article. The authors have granted ASM a non-exclusive, world-wide license to publish the Supplemental Material files. Please contact the corresponding author directly for reuse.
