# Supplementary material for: A One Health comparative genomic assessment of antimicrobial-resistant Escherichia coli in dairy farms in western Canada
Source: Appl Environ Microbiol. 2026 Jan 27;92(2):e01905-25. doi: 10.1128/aem.01905-25 (PMC12915320; doi:10.1128/aem.01905-25)
Supplement: Supplemental legend — Legend for Fig. S1. [file aem.01905-25-s0002.docx]

**Figure S1**- Maximum Likelihood phylogenetic tree of 421 *E. coli* isolates collected from animal, environmental, and human wastewater samples in the Fraser Valley region of British Columbia, Canada over a two year period (2022-24). Isolates are clustered together based on single-nucleotide polymorphism (SNP) variations, with the branch length between isolates proportional to the number of SNP differences detected. The season the isolate was recovered in is indicated in the first coloured box to the right of the AMC ID, followed by sample source, as indicated in the legend in the middle. Tree B is part of tree A, and fits as indicated by the triangle in tree A.
